# Prevalence and Genotype Distribution of Human Papillomavirus Infection Among 40,613 Women: An Outpatient-Based Population Study in Kunming, Yunnan

**DOI:** 10.3389/fpubh.2022.922587

**Published:** 2022-07-18

**Authors:** Yu Zhang, Ya Xu, Ziqin Dian, Guiqian Zhang, Xin Fan, Yuan Zhao, Yi Sun

**Affiliations:** ^1^The Affiliated Hospital of Kunming University of Science and Technology, The First People's Hospital of Yunnan Province, Kunming, China; ^2^Medical School, Kunming University of Science and Technology, Kunming, China; ^3^Department of Clinical Laboratory, The First People's Hospital of Yunnan Province, Kunming, China

**Keywords:** Human Papillomavirus, prevalence, genotype, high-risk HPV, low-risk HPV, single HPV infection, dual HPV infection, multiple HPV infections

## Abstract

Human Papillomavirus (HPV) infection is one of the most common sexually transmitted infections worldwide. The current study aimed to investigate the prevalence and genotype distribution of HPV infection among outpatient-based populations. A total of 40,613 women were recruited from the First People's Hospital of the Yunnan Province. Our study retrospectively analyzed the results of cervical HPV screening among 40,613 women. The results of study showed the prevalence and genotype distribution of HPV infection was different among various outpatient-based populations, and the prevalence of HPV infection was the highest in the gynecological outpatients (26.59%), followed by reproductive gynecological outpatients (18.51%), and the prevalence of physically examined population was the lowest (8.15%). The outpatient-based population was facing a huge threat of HPV infection, especially women from the gynecology clinic. The three most common HPV genotypes were HPV-52 (4.79%), 16 (2.95%) and 58 (2.83%). However, the distribution of HPV genotype varied by populations, especially in physically examined population, the infection rate of HPV-81 ranked third among all infections with various genotypes. Two peaks of prevalence of HPV infection were observed among women under 25 years (31.93%) and over 55 years (28.55%), while the prevalence in women aged 46–55 years (20.18%) was the lowest. Our study on the prevalence and genotype distribution of HPV infection among various outpatient-based populations will provide scientific evidence for vaccination strategies of HPV and prevention and control plans of cervical cancer in Kunming area.

## Introduction

HPV is one of the most common sexually transmitted infections worldwide. The global prevalence of HPV in women with normal cervical cytology is ~11.7% (1). Most HPV infections are transient and usually cleared or suppressed within 1–2 years by cell-mediated immunity, while a small proportion of persistent infection would eventually progress to preneoplastic lesions, resulting in cancer (2, 3). Persistent infection with high-risk HPV subtype (HR-HPV) is the main cause of invasive cervical cancer, and cervical cancer is the fourth most common cancer in women worldwide, with ~570,000 cases and 311,000 deaths in 2018, among them, there were 106,000 cases and 48,000 deaths in China, which contributed approximately one fifth of the global burden of cervical cancer (4, 5). Moreover, HR-HPV also contributes to a variable fraction of oropharyngeal, vulvar, vaginal, anal, penile, head and neck cancers, and ~5% of all cancers worldwide are associated with HR-HPV infection (6). HPV-based screening is one of the main strategies to prevent most cancers induced by HPV, especially cervical cancer (7). To the best of our knowledge, the major target groups in routine HPV-based screening in the hospital are women from the gynecology or reproductive gynecology clinic, and physical examination center. In general, most gynecological outpatients who underwent routine HPV-based screening suffered from different gynecopathy, including vaginitis, urethritis, cervicitis, cervical intraepithelial neoplasia, genital warts or undiagnosed abdominal pain, while women with infertility or underwent pre-pregnancy physical examination were more common in population screened for HPV from reproductive gynecology clinic.

A meta-analysis showed that the overall prevalence of HR-HPV among women in mainland China is 19.0% and the five most common HR-HPV subtypes are 16, 52, 58, 53, and 18 (8). However, the prevalence and genotype distribution of HPV are region-specific and age-specific. Kunming, the capital of Yunnan Province in Southwest China, has a resident population of 8.46 million. It is necessary to investigate the prevalence and genotype distribution of HPV among the various target groups, it will contribute to a more comprehensive assessment of epidemiological characteristics of HPV infection in the hospital-based population. However, to the best of our knowledge, there is a lack of studies on prevalence and genotype distribution of HPV infection covering various outpatient-based population. A better understanding will be beneficial to provide a more scientific basis for developing the prevention and control strategies of cervical cancer in Kunming area.

In addition, from July 2017 to the end of 2018, bivalent, quadrivalent, and nine-valent HPV vaccines were successively launched in China (9). However, the high cost of vaccines and shortage of vaccine supply make the improvement of vaccine coverage very difficult, especially in rural areas (10). The prevalence and genotype distribution of HPV infection was different among various populations; thus, our study investigated the epidemiological characteristics of HPV infection from outpatient-based population, including gynecology clinic, reproductive gynecology clinic, and physical examination center. First of all, it is of great significance to investigate the prevalence and genotype distribution of HPV in the hospital-based population prior to mass HPV vaccination, and it will be advantageous to establish the genotypic spectrum of HPV infection and formulate the best vaccine protection strategies for the specific target groups. Secondly, it will contribute to a more comprehensive assessment of epidemiological characteristic of HPV infection and provide a more scientific basis for developing the prevention and control strategies of cervical cancer in the local region.

Therefore, the objective of the current study was to investigate the epidemiological characteristics of HPV infection from outpatient-based population, including physical examination center, gynecology clinic, and reproductive gynecology clinic. Our study retrospectively analyzed the results of cervical HPV screening in 40,613 outpatients. We aimed to provide a scientific basis for the HPV screening and vaccination strategies in the certain populations by comparing the prevalence and genotype distribution of HPV infection in various outpatient-based populations.

## Materials and Methods

### Study Population

A total of 40,613 women who underwent HPV-based screening from the physical examination center, gynecology clinic, and reproductive gynecology clinic of the First People's Hospital of Yunnan Province were included from January 2020 to December 2021. The inclusion criteria were as follows: (1) was physically and mentally competent; (2) was a history of sexual activity at any age; (3) was willing to undergo cervical HPV screening and provided agreement to participate in the present study; (4) had not had sexual intercourse, nor used vaginal drugs in the previous 48 h (11). The exclusion criteria were as follows: (1) patients with other infections or autoimmune diseases; (2) pregnant women; (3) patients who had undergone immunosuppressive therapy (11). This research was approved by the Institutional Review Board of the First People's Hospital of Yunnan Province, and carried out strictly in accordance with the Declaration of Helsinki, maintaining the confidentiality and anonymity. Written informed consent was obtained from all the participants, and data were stored and analyzed anonymously.

### Cervical Sample Collection

Exfoliated cervical cells were collected with a cervical brush and stored at 4°C in sterile sample tubes containing cell preservation solution (HEALTH Gene Technologies Co., Ltd., Ningbo, China).

### DNA Extraction

DNA was extracted within 24 h of sample collection by using a nucleic acid extraction kit (HEALTH Gene Technologies Co., Ltd., Ningbo, China) following the manufacturer's protocol. Briefly, samples containing cervical exfoliated cells in cell preservation solution were shaken using a vortex mixer, transferred into a 1.5 ml sterile tube, and centrifuged at 12,000 g for 5 min. The supernatant was discarded and the pellet containing the cells was washed with 500 μL of PBS to remove impurities, including blood or mucus. Finally, the pellet was lysed with 200 μL of 5% Chelex-100 chelating resin for 15 min at 100 °C and centrifuged at 12,000 g for 5 min to obtain the nucleic acid.

### HPV Genotyping

HPV genotyping was performed using an HPV Genotyping Kit (HEALTH Gene Technologies Co., Ltd., Ningbo, China) following the manufacturer's protocol. Briefly, specific primers were designed for early genes E6, E7, and E1 of HPV genome, plasmid pcDNA 3.1(+), and human β-globin locus (12). The PCR amplification reaction, with the reaction mixture containing DNA template (9 μL), PCR Master Mix (9 μL) and Taq DNA polymerase (2 μL), was performed in a Veriti thermal cycler (Applied Biosystems, Foster City, CA, USA) using the following program: 5 min at 42°C, 8 min of pre-denaturation at 94°C, 35 cycles of 94°C for 30 s, 60°C for 30 s, 70°C for 1 min, with a final 1 min extension step at 70°C. Then, PCR amplification products were analyzed by using a 3500DX Genetic Analyzer (Applied Biosystems, Foster City, CA, USA), which can identify 25 HPV subtypes by capillary electrophoresis in a single analysis according to the length of PCR products, including 15 HR-HPVs (16, 18, 31, 33, 35, 39, 45, 51, 52, 56, 58, 59, 68, 73, and 82), 3 probable HR-HPVs (26, 53, and 66), and 7 LR-HPVs (6, 11, 42, 43, 44, 81, and 83). The amplification of pcDNA and human β-globin was performed to monitor the PCR process and confirm that the negative results were not due to sample insufficiency.

### Statistical Analysis

The overall, population-specific, age-specific and genotype-specific prevalence of HPV infection were calculated, respectively. Differences in the prevalence of HPV and distribution of infection patterns among different outpatient groups and age groups were analyzed using Chi-square test. All statistical analyses were performed by using SPSS statistical software, version 25.0 (SPSS, Inc., Chicago, USA). Statistical significance was set at *P* < 0.05.

## Results

### Overall Prevalence of HPV Infection

A total of 40,613 women who met the inclusion criteria were included in the current study. Among them, 27,149 (66.85%) were from the gynecology clinic (GC for short), 6,094 (15.01%) were from the reproductive gynecology clinic (RGC for short), and 7,370 (18.15%) were from the physical examination center (PEC for short). According to the results of cervical HPV screening, the overall prevalence of HPV infection was 22.03% (95% CI, 21.63–22.43%) and the prevalence varied by populations. The prevalence of HPV infection in GC group (26.59%; 95% CI, 26.06–27.11%) was the highest, followed by RGC group (18.51%; 95% CI, 17.53–19.49%), while the prevalence of PEC group (8.15%; 95% CI, 7.53–8.78%) was the lowest (*P* < 0.001, Table 1).

**Table 1:** Prevalence of HPV infection among various outpatient-based populations.

**Characteristic**	**Outpatient-based population [** ***n*** **, (%)]**				***P*-value^**a**^**
	**GC group**	**RGC group**	**PEC group**	**Total case**	
HPV-positive	7,218 (26.59)	1,128 (18.51)	601 (8.15)	8,947 (22.03)	<0.001
Infection pattern					
Single infection	5,196 (19.14)	881 (14.46)	529 (7.18)	6,606 (16.27)	<0.001
Dual infection	1,374 (5.06)	183 (3.00)	64 (0.87)	1,621 (3.99)	<0.001
Multiple infection (≥ 3)	648 (2.39)	64 (1.05)	8 (0.11)	720 (1.77)	<0.001
HPV genotype					
HR-HPV only	5,348 (19.70)	900 (14.77)	478 (6.49)	6,726 (16.56)	<0.001
LR-HPV only	985 (3.63)	136 (2.23)	98 (1.33)	1,219 (3.00)	<0.001
Mixed HR and LR HPV	885 (3.26)	92 (1.51)	25 (0.34)	1,002 (2.47)	<0.001
HR-HPV	8,157 (30.05)	1,221 (20.04)	556 (7.54)	9,934 (24.46)	<0.001
LR-HPV	2,089 (7.69)	249 (4.09)	125 (1.70)	2,463 (6.06)	<0.001

*HR, high-risk HPV; LR, low-risk HPV; GC, gynecology clinic; RGC, reproductive gynecology clinic; PEC, physical examination center*.

^a^*Comparison of three outpatient-based populations*.

### Prevalence of HR-HPV and LR-HPV Infections

The overall infection rates of HR-HPV and LR-HPV were 24.46% (95% CI, 24.04–24.88%) and 6.06% (95% CI, 5.83–6.30%), respectively. The infection rate of HR-HPV was the highest in GC group (30.05%; 95% CI, 29.50–30.60%), followed by RGC group (20.04%; 95% CI, 19.03–21.04%), while the infection rate of PEC group (7.54%; 95% CI, 6.94–8.15%) was the lowest. Moreover, a similar result was also observed for the infection rate of LR-HPV (7.69, 4.09, and 1.70%, respectively) (*P* < 0.001, Table 1).

The infection rates of HR-HPV only, LR-HPV only and mixed of HR and LR HPV were 16.56% (95% CI, 16.20–16.92%), 3.00% (95% CI, 2.84–3.17%), and 2.47% (95% CI, 2.32–2.62%), respectively. There was a significant difference in the distribution of HR-HPV only, LR-HPV only, and mixed of HR and LR HPV infections among various populations. Specifically, the infection rates of HR-HPV only (19.70%; 95% CI, 19.23–20.17%), LR-HPV only (3.63%; 95% CI, 3.41–3.85%) and mixed of HR and LR HPV (3.26%; 95% CI, 3.05–3.47%) were still the highest in GC group, followed by RGC group (14.77, 2.23, and 1.51%, respectively), and the infection rates of PEC group were still the lowest (6.49, 1.33, and 0.34%, respectively) (*P* < 0.001, Table 1).

### Prevalence of Single and Multiple HPV Infections

The overall prevalence of single infection was 16.27%, accounting for 73.83% of the total HPV-positive cases, while the prevalence of dual and multiple infections was much lower (3.99 and 1.77%, respectively), accounting for 18.12 and 8.05% of the total HPV-positive cases, respectively (Supplementary Table 1). The predominant infection pattern of HPV was single infection, while dual and multiple infections were less common. There was a significant difference in the distribution of single, dual and multiple infections in various populations. Specifically, the prevalence of single, dual and multiple infections was all the highest in GC group (19.14, 5.06, and 2.39%, respectively), followed by RGC group (14.46, 3.00, and 1.05%, respectively), and the prevalence of PEC group was all the lowest (7.18, 0.87, and 0.11%, respectively) (*P* < 0.001, Table 1).

**Figure 1:**
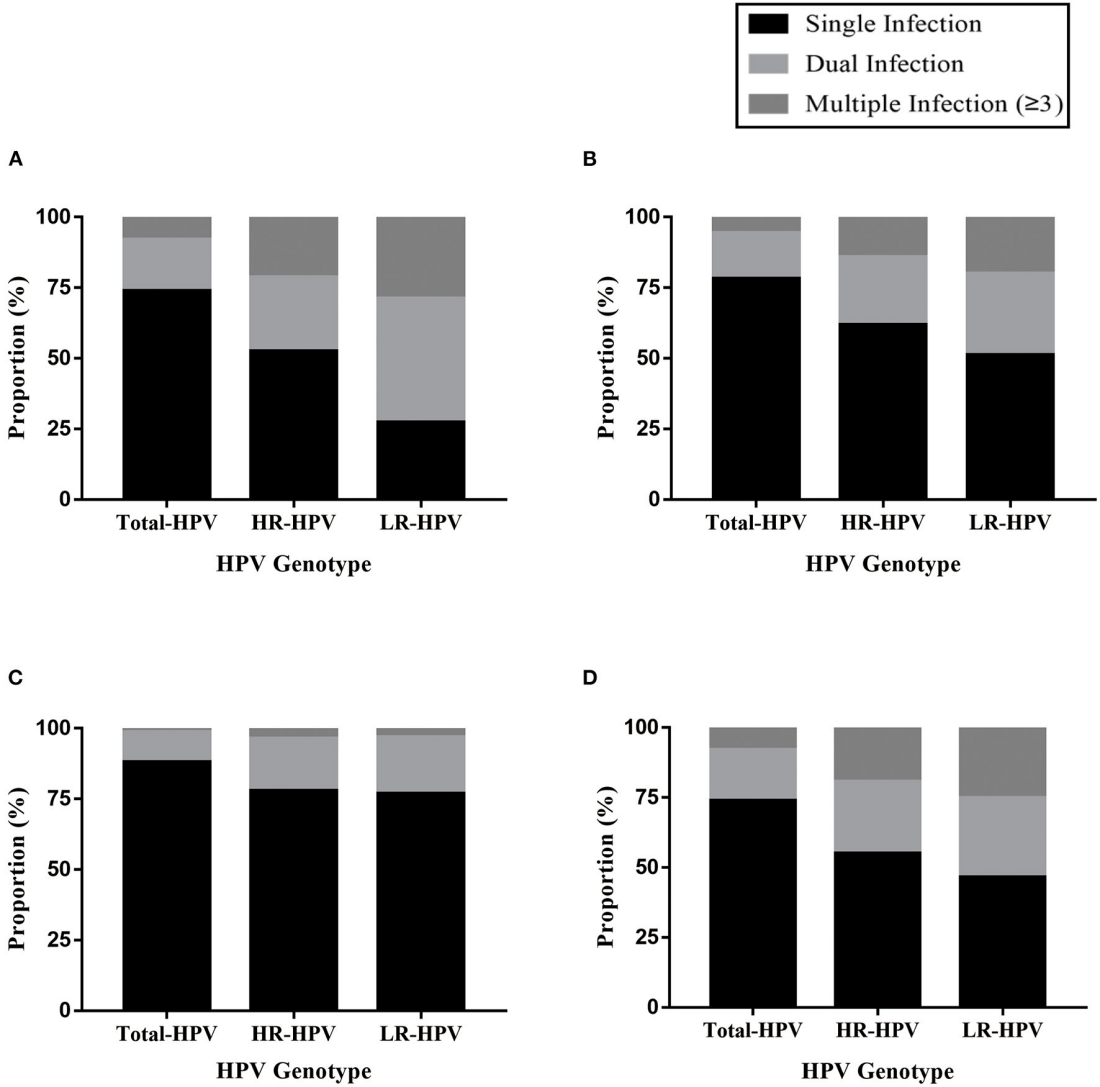
**(A)** Distribution of single and multiple infections in HPV genotypes among gynecological outpatients. **(B)** Distribution of single and multiple infections in HPV genotypes among reproductive gynecological outpatients. **(C)** Distribution of single and multiple infections in HPV genotypes among physically examined population. **(D)** Distribution of single and multiple infections in HPV genotypes among HPV-positive cases.

In addition, among the outpatient with HR-HPV infection, the proportion of single infection was the highest, accounting for 55.00%, while dual and multiple infections accounted for 25.60 and 19.40%, respectively. Even among the outpatient with LR-HPV infection, the proportion of single infection was the highest, accounting for 46.4%, while dual and multiple infections accounted for 28.4 and 25.3%, respectively. Single infection was found in 71.99% of HPV-positive cases from the GC group, accounting for 52.43% of the HR-HPV group and 43.99% of the LR-HPV group. Moreover, single infection was found in 78.10% of HPV-positive cases from the RGC group, accounting for 61.75% of the HR-HPV group and 51.00% of the LR-HPV group. Single infection was found in 88.02% of HPV-positive cases from the PEC group, accounting for 77.88% of the HR-HPV group and 76.80% of the LR-HPV group. Therefore, the proportion of single infection among HPV-positive cases was the highest in PEC group, although the prevalence of single infection was the lowest in PEC group as compared to other groups (Supplementary Tables 1–4; Figure 1).

### Distribution of HPV Genotype in HPV-Positive Population

**Figure 2:**
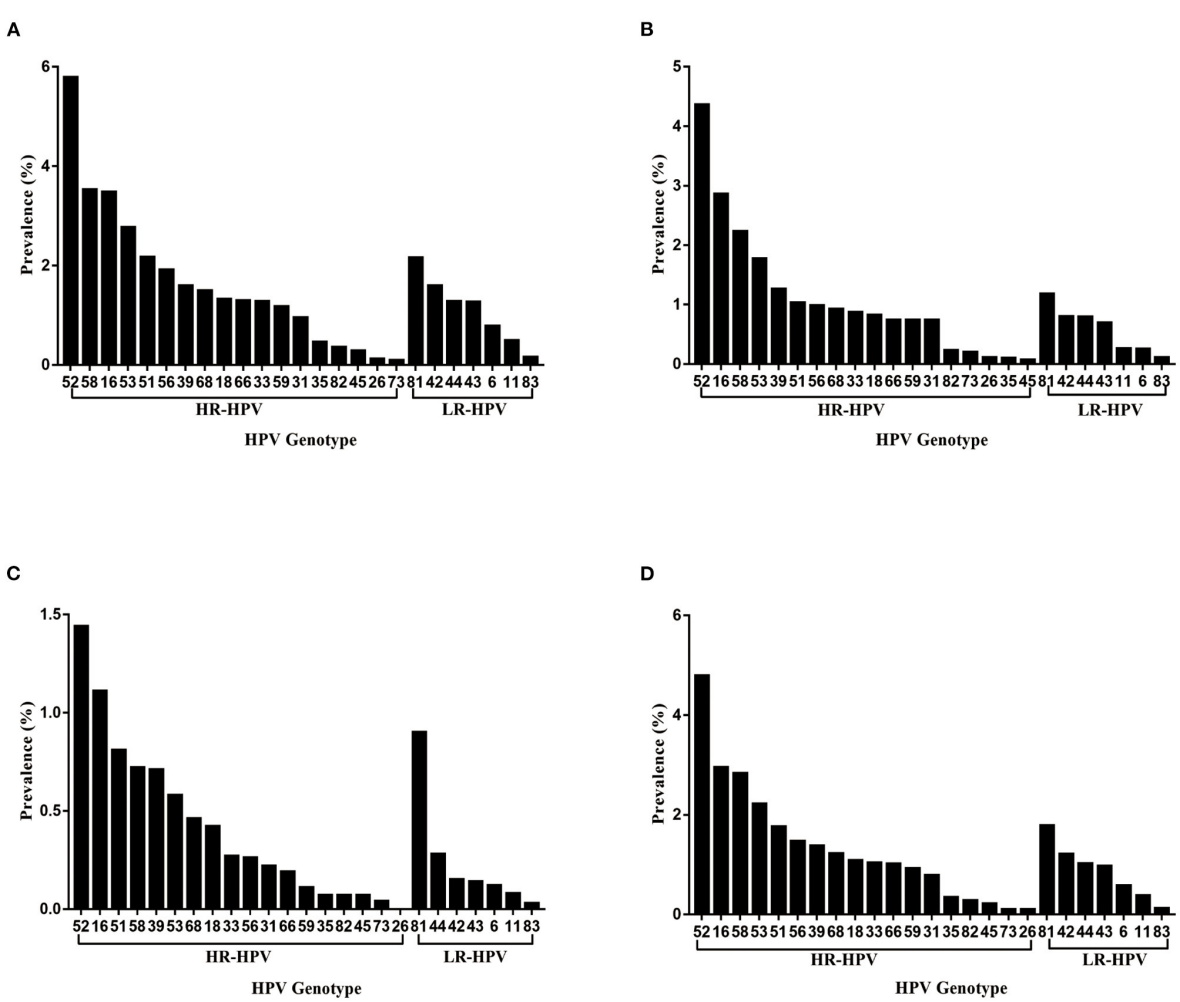
**(A–D)** Distribution of HPV genotypes among gynecological outpatients **(A)**, reproductive gynecological outpatients **(B)**, physically examined population **(C)**, total HPV-positive cases **(D)**.

The distribution of HPV genotypes among HPV-positive cases was shown in Table 2 and Figure 2. The three most common HR-HPV genotypes were HPV-52, 16 and 58 (4.79, 2.95, and 2.83%, respectively), accounting for 15.68, 9.68, and 9.26% of the total HPV-positive cases, respectively. The distribution of HPV genotype varied by populations. Specifically, in GC group, the three most prevalent HR-HPV genotypes were HPV-52 (5.79%), 58 (3.53%) and 16 (3.48%), followed by HPV-53 (2.77%) and 51 (2.17%). In RGC group, the three most prevalent HR-HPV genotypes were HPV-52 (4.36%), 16 (2.86%) and 58 (2.23%), followed by HPV-53 (1.77%) and 39 (1.26%). In PEC group, the three most common HR-HPV genotypes were HPV-52 (1.44%), 16 (1.11%) and 51 (0.81%), followed by HPV-58 (0.72%) and 39 (0.71%). In addition, the most common LR-HPV genotype was HPV-81 (1.78%) in HPV-positive cases. Notably, the infection rate of HPV-81 genotype in physically examined population was 0.90%, and be second only to HPV-52 (1.44%) and 16 (1.11%).

**Table 2:** Prevalence of HPV genotypes in HPV-positive cases.

**Genotype**	**Outpatient-based population [** ***n*** **, (%)]**								***P*-value^**b**^**
	**GC group**		**RGC group**		**PEC group**		**Total case**		
	**Prevalence**	**Proportion^**a**^**	**Prevalence**	**Proportion^**a**^**	**Prevalence**	**Proportion^**a**^**	**Prevalence**	**Proportion^**a**^**	
HR-HPVs									
HPV-52	1,572 (5.79)	15.34	266 (4.36)	18.1	106 (1.44)	15.57	1,944 (4.79)	15.68	<0.001
HPV-16	944 (3.48)	9.21	174 (2.86)	11.84	82 (1.11)	12.04	1,200 (2.95)	9.68	<0.001
HPV-58	959 (3.53)	9.36	136 (2.23)	9.25	53 (0.72)	7.78	1,148 (2.83)	9.26	<0.001
HPV-53	751 (2.77)	7.33	108 (1.77)	7.35	43 (0.58)	6.31	902 (2.22)	7.28	<0.001
HPV-51	590 (2.17)	5.76	63 (1.03)	4.29	60 (0.81)	8.81	713 (1.76)	5.75	<0.001
HPV-56	518 (1.91)	5.06	60 (0.98)	4.08	19 (0.26)	2.79	597 (1.47)	4.82	<0.001
HPV-68	404 (1.49)	3.94	56 (0.92)	3.81	34 (0.46)	4.99	494 (1.22)	3.98	<0.001
HPV-39	431 (1.59)	4.21	77 (1.26)	5.24	52 (0.71)	7.64	560 (1.38)	4.52	<0.001
HPV-18	358 (1.32)	3.49	50 (0.82)	3.4	31 (0.42)	4.55	439 (1.08)	3.54	<0.001
HPV-33	347 (1.28)	3.39	53 (0.87)	3.61	20 (0.27)	2.94	420 (1.03)	3.39	<0.001
HPV-66	350 (1.29)	3.42	45 (0.74)	3.06	14 (0.19)	2.06	409 (1.01)	3.30	<0.001
HPV-59	319 (1.17)	3.11	45 (0.74)	3.06	8 (0.11)	1.17	372 (0.92)	3.00	<0.001
HPV-31	257 (0.95)	2.51	45 (0.74)	3.06	16 (0.22)	2.35	318 (0.78)	2.57	<0.001
HPV-35	126 (0.46)	1.23	6 (0.10)	0.41	5 (0.07)	0.73	137 (0.34)	1.11	<0.001
HPV-82	96 (0.35)	0.94	14 (0.23)	0.95	5 (0.07)	0.73	115 (0.28)	0.93	<0.001
HPV-45	77 (0.28)	0.75	4 (0.07)	0.27	5 (0.07)	0.73	86 (0.21)	0.69	<0.001
HPV-73	25 (0.09)	0.24	12 (0.20)	0.82	3 (0.04)	0.44	40 (0.10)	0.32	0.013
HPV-26	33 (0.12)	0.32	7 (0.11)	0.48	–	–	40 (0.10)	0.32	0.892
LR-HPVs									
HPV-81	585 (2.15)	5.71	72 (1.18)	4.9	66 (0.90)	9.69	723 (1.78)	5.83	<0.001
HPV-42	431 (1.59)	4.21	49 (0.80)	3.33	11 (0.15)	1.62	491 (1.21)	3.96	<0.001
HPV-44	347 (1.28)	3.39	48 (0.79)	3.27	21 (0.28)	3.08	416 (1.02)	3.36	<0.001
HPV-43	341 (1.26)	3.33	42 (0.69)	2.86	10 (0.14)	1.47	393 (0.97)	3.17	<0.001
HPV-6	211 (0.78)	2.06	15 (0.25)	1.02	9 (0.12)	1.32	235 (0.58)	1.90	<0.001
HPV-11	133 (0.49)	1.3	16 (0.26)	1.09	6 (0.08)	0.88	155 (0.38)	1.25	<0.001
HPV-83	41 (0.15)	0.4	7 (0.11)	0.48	2 (0.03)	0.29	50 (0.12)	0.40	0.026
Total	7,218 (26.59)	100	1,128 (18.51)	100	601 (8.15)	100	8,947 (22.03)	100.00	<0.001

*HR, high-risk HPV; LR, low-risk HPV; GC, gynecology clinic; RGC, reproductive gynecology clinic; PEC, physical examination center*.

^a^*Accounting for percentage of the HPV-positive cases*.

^b^*Comparison of three outpatient-based populations*.

### Prevalence of HPV Infection Among Various Age Groups

**Figure 3:**
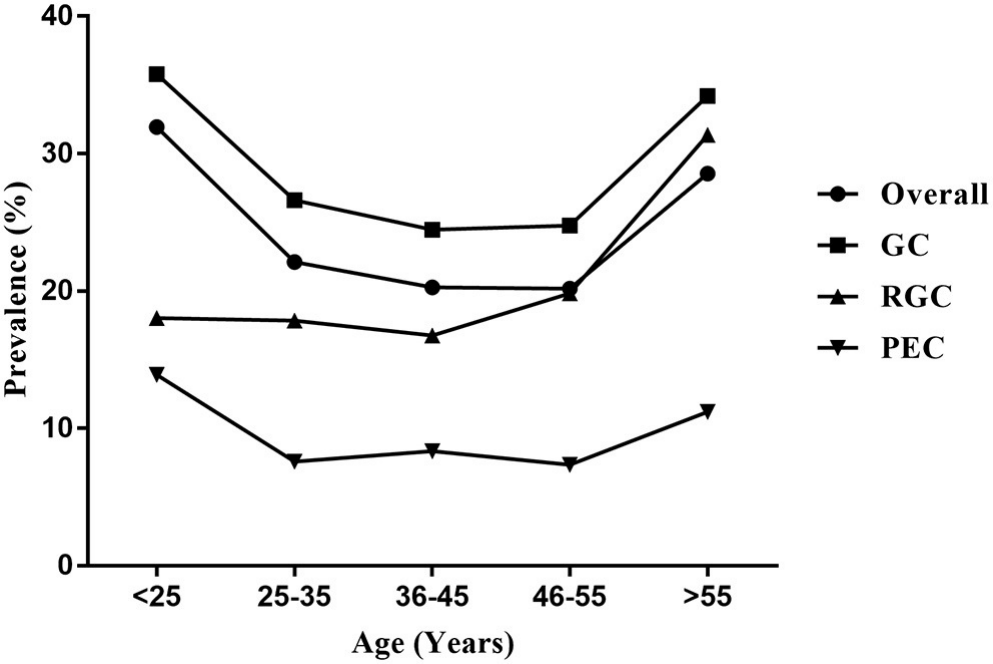
Age-specific prevalence of HPV infection in the outpatient-based population. Overall, total HPV-positive cases; GC, gynecological outpatients; RGC, reproductive gynecological outpatients; PEC, physically examined population.

The median age of the 40,613 women enrolled in the current study was 39 years (range, 16–87 years). The categories in terms of age were as follows: < 25, 25–35, 36–45, 46–55, and > 55 years. There was a significant difference in the prevalence of HPV infection among various age groups. Specifically, the prevalence of HPV infection among various age groups was 31.93, 22.12, 20.26, 20.18, and 28.55%, respectively. Two peaks of prevalence of HPV infection were observed among women under 25 years (31.93%) and over 55 years (28.55%), while the prevalence in women aged 46–55 years (20.18%) was the lowest. The prevalence of HPV infection peaked in women under 25 years, then declined with age among women aged 25–55 years, and finally peaked again in women over 55 years. Notably, the prevalence of single infection was the highest in women over 55 years (19.29%), while the prevalence of dual and multiple infections was the highest in women under 25 years (6.79 and 6.50%, respectively) (Supplementary Table 5; Figure 3).

Moreover, there was a significant difference in the prevalence of HPV infection among various populations in the same age group. The prevalence of GC group among each of age groups was all the highest (35.79, 26.60, 24.47, 24.77, and 34.20%, respectively), followed by RGC group (18.02, 17.85, 16.75, 19.83, and 31.35%, respectively). The prevalence of PEC group among each age group was all the lowest (13.89, 7.57, 8.33, 7.35, and 11.21%, respectively) (*P* < 0.001). A similar U-shaped age-specific HPV prevalence curve was observed among women from the GC or PEC, while the prevalence of HPV infection among women from the RGC did not show a U-shaped age-specific change. The prevalence was stable among women under 55 years, and significantly increased among women over 55 years in RGC group (Supplementary Tables 6–8; Figure 3).

## Discussion

HPV is one of the most common infections of the female genital tract. Prophylactic HPV vaccination is the most effective strategy for primary prevention of HPV infection. However, the immune protection of the vaccine is limited to the infection of specific subtypes, and the cross-protection effect on other subtypes is limited, the vaccine cannot provide the universal protection against HPV infection (13). The carcinogenic potential of non-vaccine-targeted HPV genotypes requires special attention due to the limited cross-protection against these genotypes. It is possible that non-vaccine-targeted HPV genotypes constitute a potential risk factor for precancerous lesions and cervical cancer among the vaccinated populations (14). Thus, it is of great significance to investigate the prevalence and genotype distribution of HPV for developing better HPV vaccination strategies and prevention and control plans for cervical cancer.

In the current study, we investigated the prevalence and genotype distribution of HPV among various outpatient-based populations in Kunming area for the first time. Although there have been previous studies on the epidemiological characteristics of HPV infection from hospital-based populations in the region, the target groups of the study by Zhang et al. were only patients who attended the department of reproductive gynecology, and the target groups in other studies were women who underwent routine gynecologic examination (15–17). However, the prevalence and genotype distribution of HPV infection was different among various populations. Therefore, our investigation covered various outpatient-based populations, including physical examination center, gynecology clinic, and reproductive gynecology clinic. The results showed the overall prevalence of HPV infection among outpatient-based population was 22.03%, and there was a significant difference in the prevalence of HPV infection among outpatients from the three departments. The prevalence of HPV infection was the highest in GC group, followed by RGC group, and the prevalence of PEC group was the lowest. Specifically, the prevalence of HPV infection was 26.59% and 8.15% in GC and PEC group, which was significantly lower than the prevalence in the neighboring Southwest Provinces of Sichuan (37.62 and 17.25%, respectively) (18). The prevalence of HPV infection was 18.51% in RGC group, which was slightly higher than the findings of Zhang et al. (15) (17.38%). Our findings showed that outpatient-based population in Kunming area was facing a huge threat of HPV infection, especially women with clinical symptoms of gynecopathy. It is possible that the presence of inflammation induces a decrease in local immunity of the genital tract, making the host more susceptible to HPV infection.

Moreover, the three most common HR-HPV genotypes among outpatient-based population were HPV-52, 16 and 58, which was in agreement with the findings of the previous studies in Kunming area (15, 17). The most prevalent HR-HPV genotype was HPV-16 in mainland China (8), while the distribution of HPV genotype varied by region, with HPV-52 being the most prevalent genotype among outpatient-based population in Kunming area. The distribution of HPV genotype also varied by populations, especially in physically examined population, the infection rate of HPV-81 ranked third among all infections with various genotypes. As one of the LR-HPV genotypes, HPV-81 is mainly associated with genital warts, its higher prevalence in physically examined population should not be underestimated (19).

The predominant pattern of HPV infection was single infection, while dual and multiple infections were less common. Several studies have reported that women co-infected with multiple HPV genotypes have a higher risk of cervical intraepithelial neoplasia and cervical cancer than those with single infection. Moreover, co-infection with multiple genotypes is associated with persistent infection and an increase in the length of the infection (20–23). We observed a higher prevalence of multiple infections in gynecological outpatients than in physically examined population, and subsequent studies are warranted to further reveal the mechanism and carcinogenic potential of multiple HPV infections and to develop special vaccination programs and prevention and control plans for cervical cancer.

With regard to the age-specific distribution of HPV infection, we found that the overall prevalence of HPV infection peaked among women under 25 years and over 55 years. The peak of HPV prevalence in younger women may be attributed to immature immune protection and higher sensitivity to infection after the beginning of sexual activity, while the peak observed in older women may be because of the reactivation of latent HPV infections due to a decrease in immune function after menopause (24). The decline in the prevalence of HPV infection among women aged 25–55 years may be attributed to mature immune protection and the fact that most infections are cleared or suppressed over time (13). Interestingly, the bimodal pattern of prevalence was absent in the outpatients from RGC, which the prevalence of HPV infection peaked only in women over 55 years. Previous studies suggest that older women with HPV infection are at a higher risk of developing persistent infection (25). Therefore, the older women should be regularly screened for cervical HPV in conditions where the HPV vaccination is not available.

In addition, we found that there was a significant increasing trend in the overall prevalence of HPV infection from 2020 to 2021 (from 20.99 to 23.02%, *P* < 0.001). No significant differences were observed in the prevalence of HPV infection between gynecological outpatients and physically examined populations during that period. But among the reproductive gynecological outpatients, there was a significant increase from 16.55% to 21.98% (*P* < 0.001). This increase may be related to the large mobility of the outpatient-based population during a certain period of time. More epidemiological data are needed to monitor the annual changes of HPV prevalence sufficiently.

The results of the current study showed the prevalence and genotype distribution of HPV infection among outpatient-based populations in Kunming area, and it will be beneficial to provide a more scientific basis for developing the prevention and control strategies of cervical cancer. However, the current study has some limitations. First, all 40,613 women were recruited from outpatient-based population, which means that our results might only represent the hospital-based populations. Second, our investigation lacked the results of cervical cytology or histology, which limited the investigation of prevalence and genotype distribution of HPV infection in women with cytology or histological abnormalities. Third, there was a lack of detailed information about the study population (such as ethnicity, educational level, and profession), so we could not evaluate the effect of these factors on the prevalence of HPV infection.

In conclusion, we conducted an outpatient-based population study on the genotype distribution and prevalence of HPV infection in Kunming area of Yunnan Province. Our results showed the prevalence and genotype distribution of HPV infection was different among various outpatient-based populations. The prevalence of HPV infection was the highest among the gynecological outpatients, followed by reproductive gynecological outpatients, and the prevalence of physically examined population was the lowest. The three most common HPV genotypes were HPV-52, 16 and 58 in the outpatient-based populations. Women under 25 years and over 55 years should pay more attention to the screening of cervical HPV. At the same time, it is necessary to actively promote the vaccination of HPV among Chinese women, especially in younger women. All of the results may be relevant in the future of prevention and control plans for cervical cancer in Kunming area.

## Data Availability Statement

The original contributions presented in the study are included in the article/Supplementary Material, further inquiries can be directed to the corresponding author/s.

## Ethics Statement

The studies involving human participants were reviewed and approved by Institutional Review Board of the First People's Hospital of Yunnan Province. The patients/participants provided their written informed consent to participate in this study.

## Author Contributions

YZhan and YX performed the collection and analysis of data and drafted the manuscript. ZD, GZ, XF, and YZhao were responsible for sample collection and HPV testing. YS conceived and supervised the project and prepared the manuscript. All authors contributed to the article and approved the submitted version.

## Funding

This project was supported by the Yunnan Health Training Project of High-Level Talents (Grant Nos. L-2019023, H-2018049, and KH-SWR-MY-2020-005), the Yunnan Provincial Department of Science and Technology-Kunming Medical University Joint Special Project [Grant No. 2019FE001 (-118)], and the Yunnan Provincial Center for Clinical Microbial Molecular Research (Grant No. 2018NS0249). The funders had no role in the study design, data collection and analysis, decision to publish, and preparation of the manuscript.

## Conflict of Interest

The authors declare that the research was conducted in the absence of any commercial or financial relationships that could be construed as a potential conflict of interest.

## Publisher's Note

All claims expressed in this article are solely those of the authors and do not necessarily represent those of their affiliated organizations, or those of the publisher, the editors and the reviewers. Any product that may be evaluated in this article, or claim that may be made by its manufacturer, is not guaranteed or endorsed by the publisher.
