# Supplementary material for: Prevalence and Genotype Distribution of Human Papillomavirus Infection Among 40,613 Women: An Outpatient-Based Population Study in Kunming, Yunnan
Source: Front Public Health. 2022 Jul 18;10:922587. doi: 10.3389/fpubh.2022.922587 (PMC9341388; doi:10.3389/fpubh.2022.922587)
Supplement: Supplementary file 1 [file Data_Sheet_1.docx]

Table S1. Distribution of single and multiple infections in HPV genotypes among total HPV-positive cases

| Group | Single Infection | | Dual Infection | | Multiple Infection (≥3) | | Total |
| --- | --- | --- | --- | --- | --- | --- | --- |
|  | Prevalence | Proportion | Prevalence | Proportion | Prevalence | Proportion |  |
| HR-HPV | 5464 (13.45) | 55.00 | 2543 (6.26) | 25.60 | 1927 (4.74) | 19.40 | 9934 (24.46) |
| LR-HPV | 1142 (2.81) | 46.37 | 699 (1.72) | 28.38 | 622 (1.53) | 25.25 | 2463 (6.06) |
| Total | 6606 (16.27) | 73.83 | 1621 (3.99) | 18.12 | 720 (1.77) | 8.05 | 8947 (22.03) |

Notes: HR-HPV, high-risk HPV; LR-HPV, low-risk HPV.

Table S2. Distribution of single and multiple infections in HPV genotypes among gynecological outpatients.

| Group | Single Infection | | Dual Infection | | Multiple Infection (≥3) | | Total |
| --- | --- | --- | --- | --- | --- | --- | --- |
|  | Prevalence | Proportion | Prevalence | Proportion | Prevalence | Proportion |  |
| HR-HPV | 4277 (15.75) | 52.43 | 2146 (7.90) | 26.31 | 1734 (6.39) | 21.26 | 8157 (30.05) |
| LR-HPV | 919 (3.39) | 43.99 | 602 (2.22) | 28.82 | 568 (2.09) | 27.19 | 2089 (7.69) |
| Total | 5196 (19.14) | 71.99 | 1374 (5.06) | 19.04 | 648 (2.39) | 8.98 | 7218 (26.59) |

Notes: HR-HPV, high-risk HPV; LR-HPV, low-risk HPV.

Table S3. Distribution of single and multiple infections in HPV genotypes among reproductive gynecological outpatients.

| Group | Single Infection | | Dual Infection | | Multiple Infection (≥3) | | Total |
| --- | --- | --- | --- | --- | --- | --- | --- |
|  | Prevalence | Proportion | Prevalence | Proportion | Prevalence | Proportion |  |
| HR-HPV | 754 (12.37) | 61.75 | 294 (4.82) | 24.08 | 173 (2.84) | 14.17 | 1221 (20.04) |
| LR-HPV | 127 (2.08) | 51.00 | 72 (1.18) | 28.92 | 50 (0.82) | 20.08 | 249 (4.09) |
| Total | 881 (14.46) | 78.10 | 183 (3.00) | 16.22 | 64 (1.05) | 5.67 | 1128 (18.51) |

Notes: HR-HPV, high-risk HPV; LR-HPV, low-risk HPV.

Table S4. Distribution of single and multiple infections in HPV genotypes among physically examined population

| Group | Single Infection | | Dual Infection | | Multiple Infection (≥3) | | Total |
| --- | --- | --- | --- | --- | --- | --- | --- |
|  | Prevalence | Proportion | Prevalence | Proportion | Prevalence | Proportion |  |
| HR-HPV | 433 (5.88) | 77.88 | 103 (1.40) | 18.53 | 20 (0.27) | 3.60 | 556 (7.54) |
| LR-HPV | 96 (1.30) | 76.80 | 25 (0.34) | 20.00 | 4 (0.05) | 3.20 | 125 (1.70) |
| Total | 529 (7.18) | 88.02 | 64 (0.87) | 10.65 | 8 (0.11) | 1.33 | 601 (8.15) |

Notes: HR-HPV, high-risk HPV; LR-HPV, low-risk HPV.

Table S5. Age-specific prevalence of HPV infection among total HPV-positive cases

| Characteristic | Age-Specific, years [n, (%)] | | | | | Total |
| --- | --- | --- | --- | --- | --- | --- |
|  | <25 | 25-35 | 36-45 | 46-55 | >55 |  |
| HPV Infection |  |  |  |  |  |  |
| HPV positive | 555 (31.93) | 3073 (22.12) | 2494 (20.26) | 1912 (20.18) | 913 (28.55) | 8947 (22.03) |
| HPV negative | 1183 (68.07) | 10821 (77.88) | 9816 (79.74) | 7561 (79.82) | 2285 (71.45) | 31666 (77.97) |
| Infection Pattern |  |  |  |  |  |  |
| Single infection | 324 (18.64) | 2258 (16.25) | 1939 (15.75) | 1468 (15.50) | 617 (19.29) | 6606 (16.27) |
| Dual infection | 118 (6.79) | 568 (4.09) | 421 (3.42) | 334 (3.53) | 180 (5.63) | 1621 (3.99) |
| Multiple infection (≥3) | 113 (6.50) | 247 (1.78) | 134 (1.09) | 110 (1.16) | 116 (3.63) | 720 (1.77) |
| Total | 1738 (4.28) | 13894 (34.21) | 12310 (30.31) | 9473 (23.33) | 3198 (7.87) | 40613 |

Table S6. Age-specific prevalence of HPV infection among gynecological outpatients.

| Characteristic | Age-Specific, years [n, (%)] | | | | | Total |
| --- | --- | --- | --- | --- | --- | --- |
|  | <25 | 25-35 | 36-45 | 46-55 | >55 |  |
| HPV Infection |  |  |  |  |  |  |
| HPV positive | 490 (35.79) | 2389 (26.60) | 2022 (24.47) | 1583 (24.77) | 734 (34.20) | 7218 (26.59) |
| HPV negative | 879 (64.21) | 6592 (73.40) | 6240 (75.53) | 4808 (75.23) | 1412 (65.80) | 19931 (73.41) |
| Infection Pattern |  |  |  |  |  |  |
| Single infection | 279 (20.38) | 1698 (18.91) | 1548 (18.74) | 1195 (18.70) | 476 (22.18) | 5196 (19.14) |
| Dual infection | 103 (7.52) | 473 (5.27) | 358 (4.33) | 287 (4.49) | 153 (7.13) | 1374 (5.06) |
| Multiple infection (≥3) | 108 (7.89) | 218 (2.43) | 116 (1.40) | 101 (1.58) | 105 (4.89) | 648 (2.39) |
| Total | 1369 (5.04) | 8981 (33.08) | 8262 (30.43) | 6391 (23.54) | 2146 (7.90) | 27149 |

Table S7. Age-specific prevalence of HPV infection among reproductive gynecological outpatients

| Characteristic | Age-Specific, years [n, (%)] | | | | | Total |
| --- | --- | --- | --- | --- | --- | --- |
|  | <25 | 25-35 | 36-45 | 46-55 | >55 |  |
| HPV Infection |  |  |  |  |  |  |
| HPV positive | 60 (18.02) | 542 (17.85) | 268 (16.75) | 163 (19.83) | 95 (31.35) | 1128 (18.51) |
| HPV negative | 273 (81.98) | 2494 (82.15) | 1332 (83.25) | 659 (80.17) | 208 (68.65) | 4966 (81.49) |
| Infection Pattern |  |  |  |  |  |  |
| Single infection | 40 (12.01) | 430 (14.16) | 215 (13.44) | 127 (15.45) | 69 (22.77) | 881 (14.46) |
| Dual infection | 15 (4.50) | 84 (2.77) | 40 (2.50) | 27 (3.28) | 17 (5.61) | 183 (3.00) |
| Multiple infection (≥3) | 5 (1.50) | 28 (0.92) | 13 (0.81) | 9 (1.09) | 9 (2.97) | 64 (1.05) |
| Total | 333 (5.46) | 3036 (49.82) | 1600 (26.26) | 822 (13.49) | 303 (4.97) | 6094 |

Table S8. Age-specific prevalence of HPV infection among physically examined population

| Characteristic | Age-Specific, years [n, (%)] | | | | | Total |
| --- | --- | --- | --- | --- | --- | --- |
|  | <25 | 25-35 | 36-45 | 46-55 | >55 |  |
| HPV Infection |  |  |  |  |  |  |
| HPV positive | 5 (13.89) | 142 (7.57) | 204 (8.33) | 166 (7.35) | 84 (11.21) | 601 (8.15) |
| HPV negative | 31 (86.11) | 1735 (92.43) | 2244 (91.67) | 2094 (92.65) | 665 (88.79) | 6769 (91.85) |
| Infection Pattern |  |  |  |  |  |  |
| Single infection | 5 (13.89) | 130 (6.93) | 176 (7.19) | 146 (6.46) | 72 (9.61) | 529 (7.18) |
| Dual infection | - | 11 (0.59) | 23 (0.94) | 20 (0.88) | 10 (1.34) | 64 (0.87) |
| Multiple infection (≥3) | - | 1 (0.05) | 5 (0.20) | - | 2 (0.27) | 8 (0.11) |
| Total | 36 (0.49) | 1877 (25.47) | 2448 (33.22) | 2260 (30.66) | 749 (10.16) | 7370 |
